# Highly Cross-Linked Polyethylene in Total Hip and Knee Replacement: Spatial Distribution of Molecular Orientation and Shape Recovery Behavior

**DOI:** 10.1155/2014/808369

**Published:** 2014-08-27

**Authors:** Yasuhito Takahashi, Toshinori Masaoka, Giuseppe Pezzotti, Takaaki Shishido, Toshiyuki Tateiwa, Kosuke Kubo, Kengo Yamamoto

**Affiliations:** ^1^Department of Bone and Joint Biomaterial Research, Tokyo Medical University, 6-7-1 Nishishinjuku, Shinjuku-ku, Tokyo 160-0023, Japan; ^2^Department of Orthopaedic Surgery, Tokyo Medical University, 6-7-1 Nishishinjuku, Shinjuku-ku, Tokyo 160-0023, Japan; ^3^Ceramic Physics Laboratory, Kyoto Institute of Technology, Sakyo-ku, Matsugasaki, Kyoto 606-8585, Japan

## Abstract

The present study investigated effects of processing procedures on morphology of highly cross-linked and re-melted UHMWPE (XLPE) in total hip and knee arthroplasty (THA, TKA). The shape recovery behavior was also monitored via uniaxial compression test at room temperature after non-destructive characterizations of the in-depth microstructure by confocal/polarized Raman spectroscopy. The goal of this study was to relate the manufacturing-induced morphology to the *in vivo* micromechanical performance, and ultimately to explore an optimal structure in each alternative joint bearing. It was clearly confirmed that the investigated XLPE hip and knee implants, which were produced from different orthopaedic grade resins (GUR 1050 and GUR 1020), consisted of two structural regions in the as-received states: the near-surface transitional anisotropic layer (≈100 *μ*m thickness) and the bulk isotropic structural region. These XLPEs exhibited a different crystalline anisotropy and molecular texture within the near-surface layers. In addition, the knee insert showed a slightly higher efficiency of shape recovery against the applied strain over the hip liner owing to a markedly higher percentage of the bulk amorphous phase with intermolecular cross-linking. The quantitative data presented in this study might contribute to construct manufacturing strategies for further rationalized structures as alternative bearings in THA and TKA.

## 1. Introduction

Increased incidence of periprosthetic osteolysis with increasing polyethylene wear has historically been the leading complication of total hip arthroplasty (THA), resulting in the growing necessity of revision surgery due to aseptic loosening of implant devices [1–3]. In the late 1990s, cross-linked and thermally treated ultrahigh molecular weight polyethylene (UHMWPE), the so-called first-generation highly cross-linked polyethylene (XLPE), was hence developed for THA with expectation of the improved wear resistance over conventional UHMWPE [4–6]. The implementation of XLPE acetabular liners certainly contributed to a dramatic reduction in the volumetric wear [4–8] as well as in the oxidative degradation [9]. Nevertheless, the concomitant decrease in toughness, ultimate tensile strength, and fatigue crack propagation resistance was also recognized [9–13]. The above trade-off problem, associated with intermolecular cross-linking of C–C chemical bonds between adjacent chains, led to a subject of considerable discussion as to its application to tibial inserts used in total knee arthroplasty (TKA) [14].

The kinematics of knee joint represents more anisotropic/constrained motions within small surface area of contact, leading to higher compressive and shear stresses as compared to that of hip joint. Under the applied loading of 3000 N (the entire joint load on the component), the maximum compressive stresses at the surface of UHMWPE components were computed by finite element analysis (FEA) as ≈15 MPa and ≈40 MPa for hip (using 28 mm head) and knee joint, respectively [15]. On the other hand, the maximum shear stresses were found at the very surface for the hip but at the depth of 1-2 mm below the surface for the knee, whose values were evaluated as ≈5 MPa and ≈10 MPa, respectively [15, 16]. Moreover, it should be noted that the most common cause of revision TKA has been mechanical loosening (40%) rather than wear/osteolysis (9%) [17]. This epidemiological data may support the results of the above theoretical considerations for the biomechanical and kinematic differences between hip and knee joints. In this context, the required microstructure for each joint application should carefully be considered and optimized at the molecular scale.

Although several manufacturing attempts in industry have been made so far to rationalize preferred choices of resin types (GUR 1050 or GUR 1020), consolidation methods (sheet compression, ram extrusion, or direct compression molding), and radiation doses (50~300 kGy), consensus for alternative bearings has not been achieved yet among polymer scientists [18, 19]. A key underlying concept of balancing among the wear resistance and mechanical properties could be given by the optimization of molecular anisotropy, entanglement characteristics, and the balanced percentages between crystalline and noncrystalline phases, that is, amorphous and third intermediate phase. More specifically, the importance should be placed on minimizing the occurrence of strain-softening (strain-weakening) phenomenon [19–21] and irrecoverable plastic deformation during each characteristic* in vivo* motion of hip and knee joints. From this viewpoint, it would be of tremendous benefits to explore the effects of the manufacturing-induced local strain and plastic deformation on the initial molecular anisotropies in the final products for maximizing the potential wear performances.

The present investigation focuses on quantifying the molecular anisotropy and phase percentages such as crystalline, amorphous, and third intermediate phase along the depths of commercially available XLPE hip and knee joint prostheses by means of high spatial resolution Raman microscopy. In addition, the deformation and shape recovery behaviors from external uniaxial compressive forces were also monitored. The quantitative data obtained in this study might contribute to construct further improved manufacturing strategies for highly rationalized structures as alternative joint bearings for hip and knee arthroplasty applications and also to minimize the time-consuming manufacturing trials.

## 2. Materials and Methods

### 2.1. Study Design

The first-generation XLPE hip and knee implants were evaluated through the following quantitative analyses: (i) phase percentages, that is, amorphous, crystalline, and third intermediate phase; (ii) preferred orientation of molecular chains; (iii) degree of crystalline anisotropy; and (iv) deformation and shape recovery behaviors from applied uniaxial compression. We have also built a structural model based on the results of the above morphological characterizations in order to discuss the potential* in vivo* performance and functionality of each type of prosthesis.

### 2.2. XLPE Hip and Knee Prostheses

**Figure 1:**
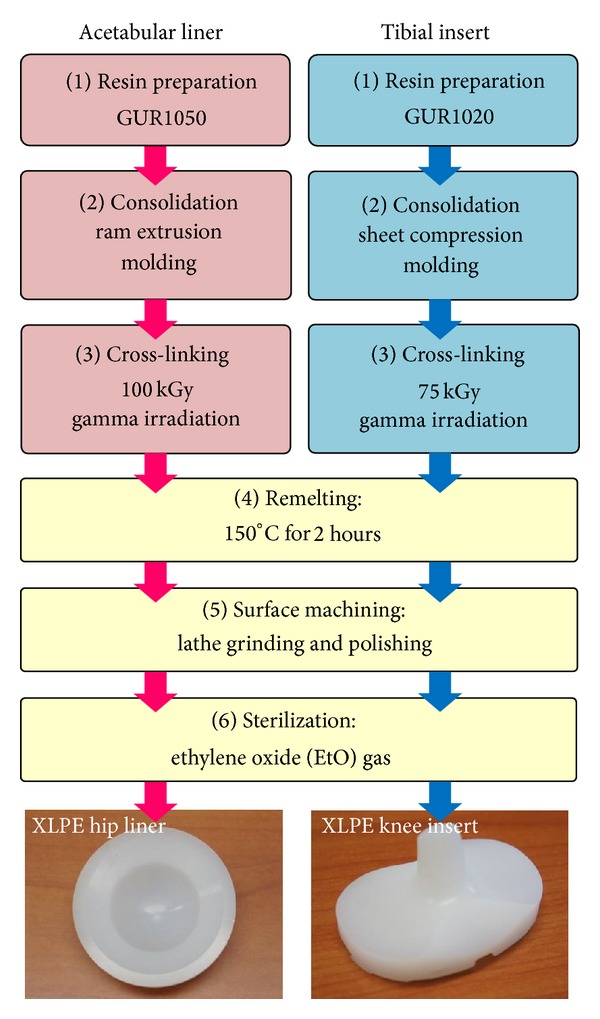
Processing methods for XLPE acetabular liner and tibial insert for total hip and knee arthroplasty.

The never implanted, first-generation remelted XLPE acetabular liner and tibial insert (*n* = 3 for each prosthesis) were analyzed in this study. The investigated hip and knee bearings are both referred to as the same trade name, XLPE, which were manufactured by Smith & Nephew Orthopedics, Inc. (Memphis, Tennessee, USA). XLPE has been clinically introduced since 2001 for THA and 2008 for TKA in USA. The thickness of these test components was 10 mm for the hip and 11 mm for the knee in posterior stabilized (PS) designs. A comparison of the processing procedures for each XLPE component was given in Figure 1. The manufacturing of XLPE hip liners starts from GUR 1050 resin (Ticona Inc., Florence, KY, USA) with a molecular weight of approximately 6.0 million g/mol. Ram-extruded GUR 1050 rods were cross-linked by gamma-ray irradiation with a total dose of 100 kGy and then remelted at 150°C for 2 hours to eliminate all the free radicals. On the other hand, XLPE knee inserts start from GUR 1020 resin (Ticona Inc., Florence, KY, USA) with a molecular weight of approximately 3.5 million g/mol. Compression-molded GUR 1020 sheets were cross-linked by gamma-ray irradiation with a total dose of 75 kGy and then remelted under the same condition (150°C for 2 hours). After being machined into the final shapes of the acetabular liners or the tibial inserts, the materials were barrier packaged and exposed to ethylene oxide (EtO) gas for the sterilization purposes.

### 2.3. Raman Spectroscopy

#### 2.3.1. Phase Volume Fractions Analyses

**Figure 2:**
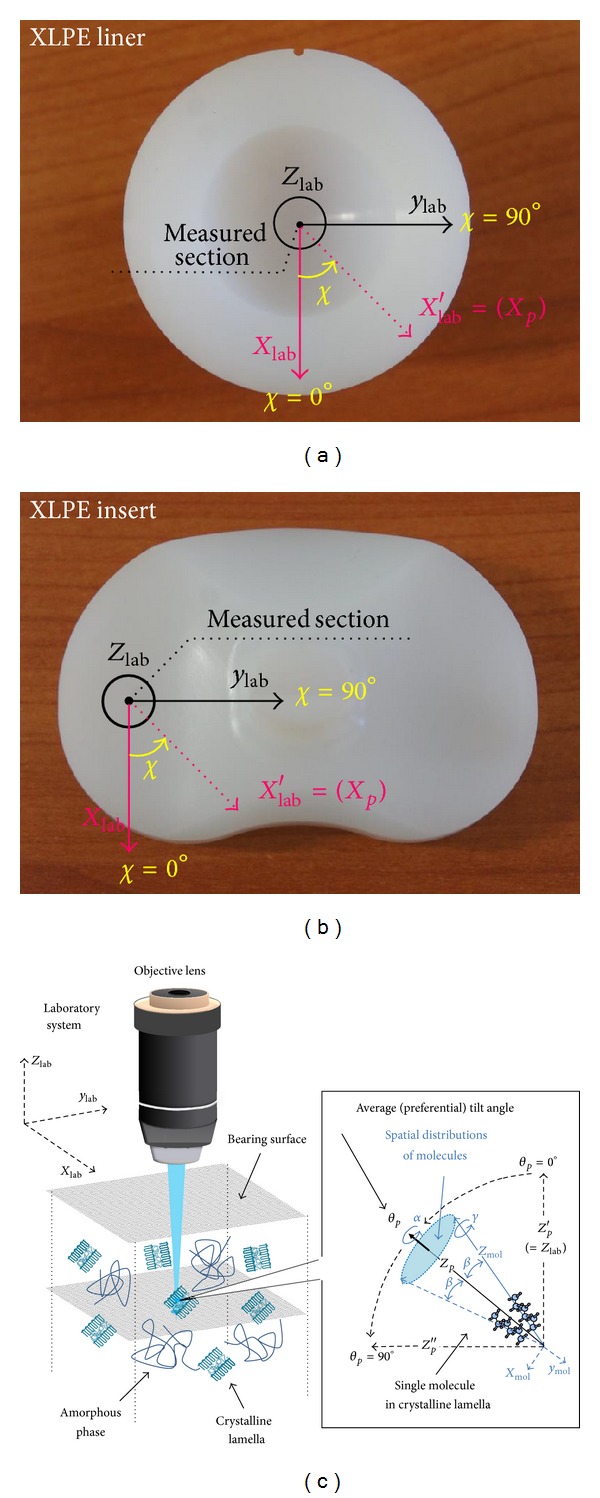
(a) Schematic of the selected measurement sections and definition of inplane rotation angle, *χ*, for XLPE hip and knee components. (b) Schematic of our choice of Cartesian reference systems and of the Euler angles governing their rotations in space, as explained in the text (Section 2.3.2).

For the phase fractional assessments of melt-like amorphous (*α*_*a*_), orthorhombic crystal (*α*_*c*_), and third intermediate region (*α*_*t*_), nondestructive Raman spectroscopic analyses were conducted. The measured sections for each test sample, where intensive plastic deformation and wear would be expected to occur during the* in vivo* services [22], were indicated in Figures 2(a)-2(b). All the spectroscopic measurements in this study were made by means of Raman microprobe spectrometer (MS3504i, SOL Instruments Ltd., Minsk, Republic of Belarus) in back-scattering geometry. The excitation source was 514.5 nm Ar-ion laser (GLG3103, Showa Optronics Co., Ltd., Tokyo, Japan) yielding a power of approximately 20 mW on the sample surfaces. The confocal configuration of the probe adopted throughout the present experiments corresponded to a ×100 objective; numerical aperture, focal length, and pinhole diameter were fixed as 0.6, 7.6 mm, and 100 *μ*m, respectively. Individual spectra were typically collected in 15 seconds. The recorded spectra were averaged over three successive measurements. The focal plane was eventually shifted toward the sample subsurface direction in order to nondestructively screen the depth regions of the samples. At each depth of samples, an inplane sampling of 2.5 *μ*m lateral steps was applied within the area of 50 × 50 *μ*m^2^(for a total of 1323 spectra per each map). A total of 2205 different locations (= 441 points/map × 5 maps) were selected within the sections, and the average value was assumed to be representative of the phase fractions at each selected depth. The computations of these fractions were made according to the following equations [23–25]:

(1) $$\begin{array}{c} \alpha_{a}=\frac{I_{1080}}{0.79\left(I_{1296}+I_{1310}\right)},\\ \alpha_{c}=\frac{I_{1418}}{0.46\left(I_{1296}+I_{1310}\right)}, \end{array}$$

where *I* is the integral intensity of the Raman band whose wavenumber is identified by the subscript. Note also that the sum (*α*_*a*_ + *α*_*c*_) might locally be <1, because of the possible presence of an anisotropic intermediate state (usually referred to as the “third phase” [26]). The volume fraction of *α*_*t*_ can be thus provided by

(2) $$\begin{array}{c} \alpha_{t}=1-\;\left(\alpha_{c}+\alpha_{a}\right). \end{array}$$

#### 2.3.2. Molecular Orientation of Carbon Chains and Anisotropy Analyses

For the assessments of three-dimensional molecular orientation of the carbon chains (the C–C alkyl chains) and the degree of crystalline anisotropy, polarized measurements combined with confocal Raman spectroscopy were conducted in the same sections where the phase percentages were examined. During the measurements, a parallel polarization filter and a half-wave plate were placed between samples and spectrometer and set to pass scattered Raman radiation horizontally to a CCD camera. To express the spatial position and distribution of the molecular orientation, three different Cartesian coordinate systems were defined as a system (*x*_lab_ *y*_lab_ *z*_lab_) describing the laboratory frame, a system (*x*_*p*_ *y*_*p*_ *z*_*p*_) to describe the average preferential molecular orientation (as detected by the Raman probe of finite dimensions), and a system (*x*_mol_ *y*_mol_ *z*_mol_) to describe the orientation of individual molecular chains with respect to the mean axes of preferential orientation. Euler angles for the above three Cartesian frames can be defined as a set of angle (*θ*, *φ*, *χ*) describing the rotation of (*x*_mol_ *y*_mol_ *z*_mol_) with respect to (*x*_lab_ *y*_lab_ *z*_lab_), a set of angle (*θ*_*p*_, *φ*_*p*_, *χ*_*p*_) describing the rotation of (*x*_*p*_ *y*_*p*_ *z*_*p*_) with respect to (*x*_lab_ *y*_lab_ *z*_lab_), and a set of angle (*α*, *β*, *γ*) describing the rotation of (*x*_mol_ *y*_mol_ *z*_mol_) with respect to (*x*_*p*_ *y*_*p*_ *z*_*p*_). Note that the (*θ*_*p*_, *φ*_*p*_, *χ*_*p*_) determines the preferential molecular orientation in the XLPE components and (*α*, *β*, *γ*) determines the spatial distribution angles from preferential orientation (*θ*_*p*_, *φ*_*p*_, *χ*_*p*_). However, taking into consideration the cylindrical symmetries of the long linear polyethylene chains, the numbers of the orientation angles relevant to Raman scattering may be reduced. In such a symmetric case, we can neglect the dependences of any torsional orientation such as *φ*, *φ*_*p*_, *α*, and *γ*. In this study, we set the angle *θ*_*p*_, that is, the out-of-plane tilt angle, as the preferred molecular orientation. Specifically, *θ*_*p*_ = 0° indicates the alignment of C–C chains perpendicular to the articulating surfaces, while *θ*_*p*_ = 90° indicates the alignment of C–C chains parallel to the surfaces. In addition, we assumed the existence of a uniaxial symmetry with respect to the preferential orientation of the molecular chains, which is only dependent on one polar angle, *β*  (Figure 2(c)); that is, angles *α* and *γ* do not enter the expression of molecular distribution. Thus, the probability of finding polyethylene molecules with orientations between *β* and (*β* + *dβ*) can be analytically formulated as follows [26]:

(3) $$\begin{array}{c} \overset{\gamma =2\pi }{\underset{\gamma =0}{\int }}\overset{\alpha =2\pi }{\underset{\alpha =0}{\int }}\overset{\beta =2\pi }{\underset{\beta =0}{\int }}f\left(\beta \right)\sin\beta d\beta d\alpha d\gamma =1\left(f\left(\beta \right)\geq 0\right), \end{array}$$

where *f*(*β*) is called orientational probability distribution or orientation distribution function (ODF).

As the experimental procedures, XLPE samples were placed on a *χ*-axis (inplane angle on the bearing surface) rotation jig and polarized Raman spectra in parallel configuration were collected at 19 different azimuthal angles within the interval 0 ≤ *χ* ≤ 180°, with sequential rotational steps of 10° (Figures 2(a)-2(b)). Particular care was taken in order to align the axis of the microscope with the axis of the rotation jig. It is known that the polarized Raman band located at 1130 cm^−1^ related to the C–C stretching vibration (*A*_*g*_ + *B*_1*g*_ mode) is the most affected by polyethylene orientation [27, 28] and its angular dependence was used also in this study as a sensor to examine preferential orientation and its degree of order. Considering that the observed scattering intensity represents the contribution from all the individual polyethylene molecules existing within the volume of laser probe, the overall polarized Raman intensity (*I*_1130_^||^) for XLPE products is provided by the following general equation [28, 29]:

(4) $$\begin{array}{c} I_{1130}^{||}=\frac{\int_{\gamma =0}^{\gamma =2\pi }\int_{\alpha =0}^{\alpha =2\pi }\int_{\beta =0}^{\beta =2\pi }I_{A_{g}+B_{1g}}^{||}\left(\theta ,\chi \right)f\left(\beta \right)\sin\beta d\beta d\alpha d\gamma }{\int_{\gamma =0}^{\gamma =2\pi }\int_{\alpha =0}^{\alpha =2\pi }\int_{\beta =0}^{\beta =2\pi }f\left(\beta \right)\sin\beta d\beta d\alpha d\gamma }, \end{array}$$

where *I*_*A*_*g*_+*B*_1*g*__^||^ is the dependence of Raman intensity of a single polyethylene molecular chain in parallel polarization configuration according to its angular dependence on Euler angles, *θ* and *χ* (note that *θ* and *χ* are functions of *θ*_*p*_ and *χ*_*p*_) [28, 29], and the ODF, *f*(*β*), representative of the molecules oriented in a cone around the orientation axis (cf. Figure 2(c)), will take the following form:

(5) $$\begin{array}{c} f\left(\beta \right)=A\exp\left\{-\left[\lambda_{2}P_{2}\left(\cos\beta \right)+\lambda_{4}P_{4}\left(\cos\beta \right)\right]\right\}, \end{array}$$

where *A* is a constant and the parameters *λ*_*i*_  (*i* = 2,4) are the so-called Lagrange multipliers; that is, *P*_2_  (cos⁡*β*) = (3cos⁡^2^ *β* − 1)/2, *P*_4_  (cos⁡*β*) = (35cos⁡^4^⁡*β* − 30cos⁡^2^⁡*β* + 3)/8, used in the definition of the principle of maximum information entropy [30, 31]. A mathematical procedure was performed by using (4)-(5) to find the best-fitting curves to data sets of polarized Raman intensity collected at different inplane orientation (*χ*). Setting a computational routine, *θ*_*p*_ and *f*(*β*) can be determined, and the degree of molecular orientation can be calculated using the following equation [28, 29, 31, 32]:

(6) ∫γ=0γ=2π∫α=0α=2π∫β=0β=πP2(cos⁡β)f(β)sinβdβdαdγ  =〈P2(cosβ)〉,

where 〈*P*_2_(cos⁡*β*)〉 is referred to as Herman's orientation parameter and represents the degree of molecular orientation. A value 0 for it indicates that the molecular orientation is fully random (isotropic), while a value 1 represents a perfect orientation along a preferential orientation axis. For a partial molecular orientation, the value should be 0 < 〈*P*_2_(cos⁡*β*)〉 < 1.0.

### 2.4. Compression Deformation Test

In the present investigation, a compressive deformation was applied at room temperature (24 ± 2°C) in the samples purposely prepared by cutting the unused XLPE acetabular liner and tibial insert into rectangular prisms 3 × 3 × 6 mm in dimension. These rectangular samples (*n* = 3 for each liner) were obtained from each surface of these samples where the morphological assessments were performed by means of Raman spectroscopy. Deformation tests (cross-head speed of 0.1 mm/min) were performed using uniaxial compression equipment. The rectangular specimens were obtained from the sections corresponding to the Raman investigations. Particular care was taken in order to smooth down only the four corners of the slightly concave surfaces of the samples. This latter procedure enabled us to flatten the originally concave surfaces to match the compressive surface of the jig while preserving the original microstructure of the samples, thus reproducing as closely as possible the conditions encountered during* in vivo* loading. One-dimensional stress relaxation tests were also performed. The residual strain in each as-received material was assumed as *ε* = 0 and an increasing compressive load was applied by a 3% strain (180 *μ*m) step. At each step, the materials were subjected to a compressive strain of a predetermined magnitude, which was kept constant for at least 24 hours in order to allow the full development of internal deformation allowed by the microstructure. The load was then released and the samples allowed recovering of the inelastic strain for at least 24 hours, a time interval sufficient to obtain a nearly full recovery, especially at low and moderate levels of deformation, as in the case of the present investigation [33, 34]. At each step, the engineering strain was measured along the sample long axis by means of a micrometer caliper both before and after recovery, henceforth referred to as *ε*_*i*_ and *ε*_*f*_, respectively.

## 3. Results

### 3.1. Phase Volume Fractions

**Figure 3:**
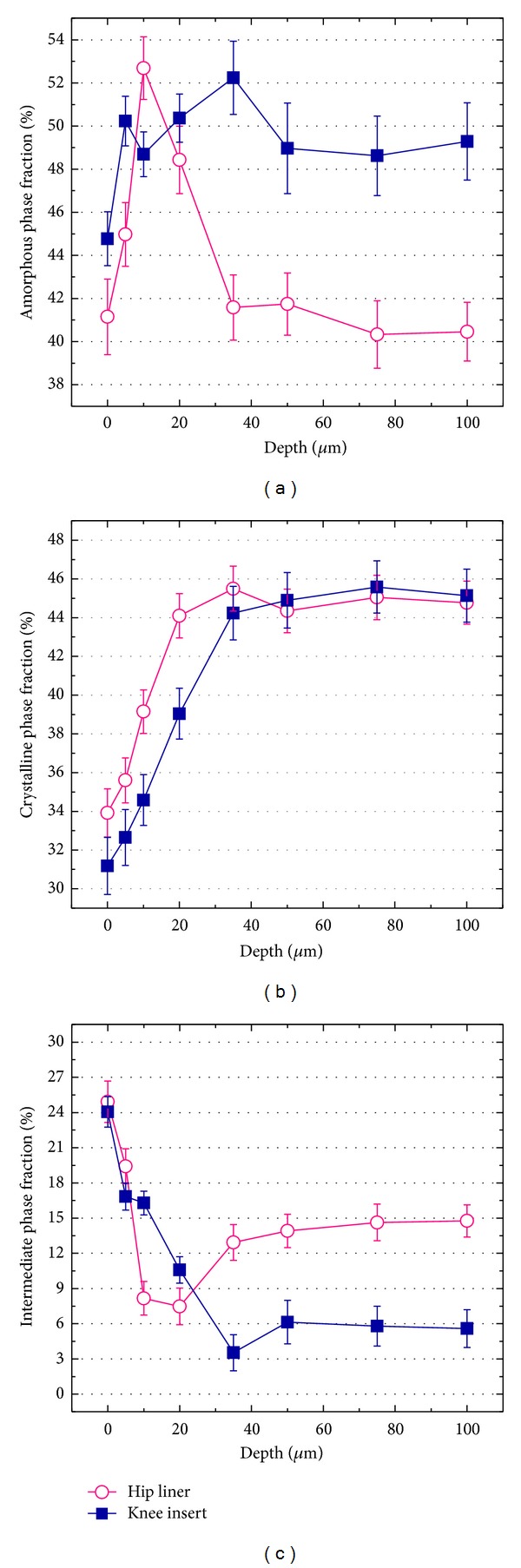
Depth profiles of phase volume fractions collected in the as-received XLPE hip and knee components ((a) amorphous fraction, *α*_*a*_, (b) crystallinity, *α*_*c*_, and (c) intermediate fraction, *α*_*t*_, resp.).

Figures 3(a)–3(c) show the depth profiles of the amorphous, crystalline, and third intermediate phase percentages (*α*_*a*_, *α*_*c*_, and *α*_*t*_, resp.) as detected in XLPE hip liner and knee insert. Confocal Raman analyses confirmed that the phase fractions in both products rapidly change along the subsurface depths within the first 35 *μ*m from the articulating surface, but subsequently show the nearly constant and homogeneous trends. The differences between minimum and maximum values along the *α*_*c*_ and *α*_*t*_ profiles (Figures 3(b) and 3(c)) were larger in the knee insert; that is, Δ*α*_*c*_ and Δ*α*_*t*_ were 14.0% and 19.3% for the knee and 10.9% and 17.4% for the hip liner, respectively. Although GUR 1020, used as the starting resin for manufacturing the knee insert, initially possesses a higher *α*_*c*_ value before gamma irradiation and remelting as compared to GUR 1050 resin used for the hip liner [35], a similar percentage of the bulk crystallinity (*α*_*c*_ = 45.04 ± 1.15% and 45.13 ± 1.37% in the hip and knee, resp.) was observed in their final products. On the other hand, their bulk profiles exhibited about 9% difference in the *α*_*a*_ and *α*_*t*_. XLPE knee showed a markedly higher amorphous phase percentage, that is, a lower third intermediate phase content, in its bulk region, as compared to the hip.

### 3.2. Molecular Orientation of Carbon Chains

**Figure 4:**
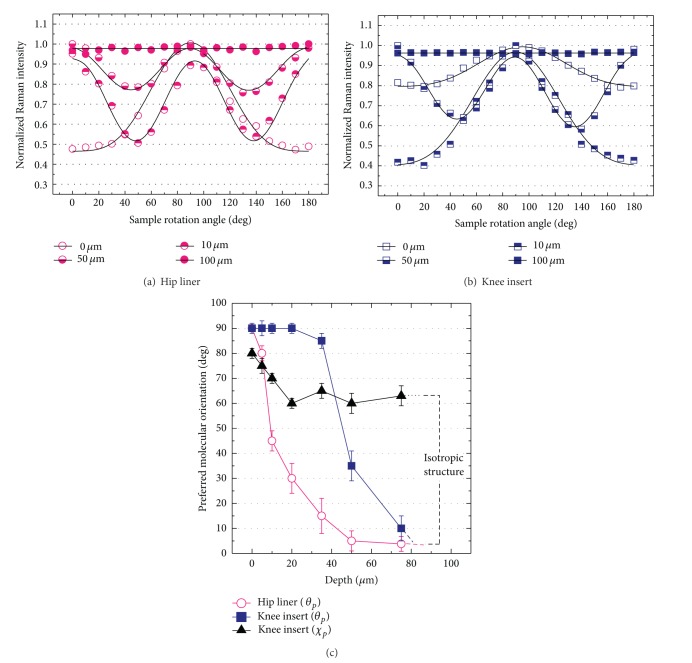
Experimental plots of the angular dependence of polarized Raman scattering intensities recorded upon inplane rotation *χ* at different depth of the as-received XLPE hip (a) and knee (b) components. Full lines represent the results of best fitting to the experimental data according to (4) in Section 2.3.2. Depth profiles of preferential orientation of the carbon chains (the C–C alkyl chains) collected in the as-received XLPE hip and knee components (c).

The normalized Raman intensities (*I*_1130_^||^) of XLPE hip and knee implants depending on the inplane sample rotation angle (*χ*) were plotted in Figures 4(a) and 4(b), respectively. According to the least-square method, the best-fitting curves determined using (4)-(5) with minimum deviations from the data points were also plotted in the figures. The good agreement between theoretical curves and experimental plots was confirmed, indicating the high degree of accuracy and reproducibility of the results. The variation of the polarized Raman intensity depending on the rotation angle is pronounced when the sample possesses a high degree of anisotropy. In addition, the *π* or *π*/2 angular periodicities of *I*_1130_^||^ plots are indicative of a preferred molecular orientation (*θ*_*p*_) nearly parallel or perpendicular to the articulating surfaces, respectively, while the constant intensity (no angular periodicity) trend implies the isotropic molecular structure (no preferred orientation).

The depth profiles of the *θ*_*p*_ angle obtained from the data sets of Figures 4(a)-4(b) were given in Figure 4(c). As explained in Section 2.3.2, *θ*_*p*_ = 0° represents the C–C chain alignment perpendicular to the sample surface, while *θ*_*p*_ = 90° indicates the chain alignment parallel to the surface. The C–C chains at the superficial layers of the hip and knee components preferentially lie in a direction parallel to each surface and subsequently tend to reach a direction nearly perpendicular to the surface with proceeding along the subsurface depths. Isotropic structure appears at the depths within 75 to 100 *μ*m. There was a significant difference in the *θ*_*p*_ profiles within the first 50 *μ*m depth between the two prostheses. The *θ*_*p*_ angles of the hip liner more rapidly decrease from the surface down to subsurface than the knee insert. In other words, the knee insert possesses a thicker layer of parallel molecular orientation in its near-surface region.

In addition to the tilt angle of *θ*_*p*_, the preferred inplane orientation angle, *χ*_*p*_, also can be obtained simultaneously in the same fitting procedures. However, this angle apparently cannot be identified in the hip acetabular liner due to its rotationally symmetric shape (cf.  Figure 2(a)). Thus, the depth profile of *χ*_*p*_ was given only for the knee insert in Figure 4(c). The C–C chains were preferentially oriented at an angle of *χ*_*p*_ = 80° ± 2°, which represents the molecules oriented at 10° away from the medial-lateral axis of the knee components in a clockwise direction. It was also confirmed that the surface and subsurface angles were slightly twisted (Δ*χ*_*p*_ ≤ 20°) within the depth of about 75 *μ*m and the C–C chains gradually rotated toward the anterior-posterior axis with proceeding along the depth.

### 3.3. Orientational Probability Distribution and Degree of Anisotropy

**Figure 5:**
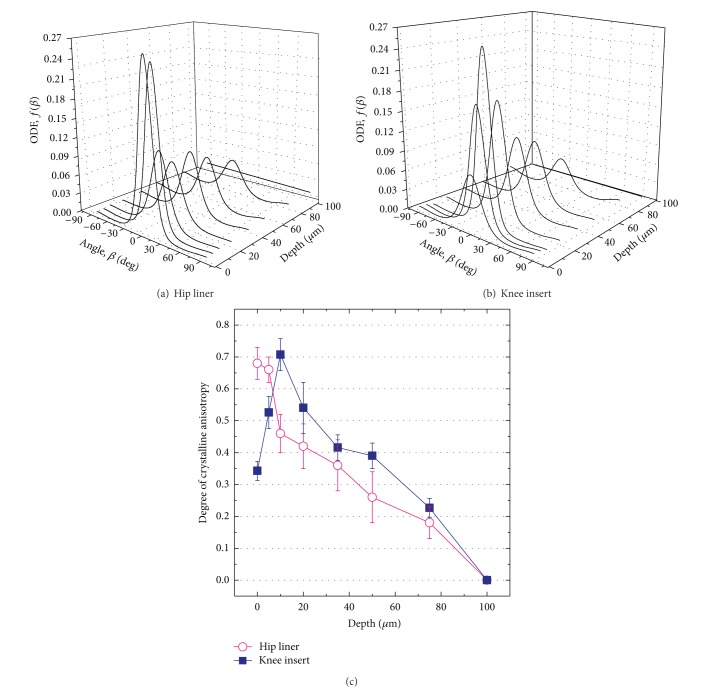
Orientation distribution functions (ODFs, *f*(*β*)) calculated at the different depth of the as-received XLPE hip (a) and knee (b) components. Depth profiles of degree of crystalline anisotropy, 〈*P*_2_(cos⁡*β*)〉 collected in the as-received XLPE hip and knee components (c).

Figures 5(a)-5(b) show the orientation distribution functions (ODFs, *f*(*β*)) as determined at each depth of the hip and knee samples. The angle *β* represents orientation of individual molecular chains with respect to the mean preferential orientation (*θ*_*p*_). Note that the molecular axis of *β* = 0 corresponds to the axis of *θ*_*p*_. Therefore, a material with a higher anisotropy shows a narrower (shaper) shaped curve of ODF, while a lower anisotropic material represents a broader (flatter) ODF.

In the superficial layers of both components, quite different ODFs were obtained. In the hip liner, significantly narrow distributions of C–C chain orientation were found within the first 5 *μ*m below the surface, while ODFs for the knee start from a much broader distribution at the surface and then rapidly increase up to the subsurface depth of 10 *μ*m. Beyond the depth, both components showed a similar trend of ODFs, which were gradually getting broader with the increase in the subsurface depths.

The degree of anisotropy 〈*P*_2_(cos⁡*β*)〉 was plotted in Figure 5(c) as a function of depth in the studied implants. As already defined in the previous section, 〈*P*_2_(cos⁡*β*)〉 takes a range of values from a minimum of 0 (isotropic) to a maximum of 1.0 (perfect orientation). Figure 5(c) showed that the hip liner possessed a highly anisotropic surface, and the 〈*P*_2_(cos⁡*β*)〉 value was obtained as 0.68 ± 0.05. However, the knee surface showed a much lower value (〈*P*_2_(cos⁡*β*)〉 = 0.34 ± 0.03). The maximum values of 〈*P*_2_(cos⁡*β*)〉 were observed at the depths of 0 and 10 *μ*m for the hip and knee, respectively, and these subsequently decreased down to 0 at the depth of 100 *μ*m. In the subsurface regions from 10 to 100 *μ*m, the knee insert had a slightly higher anisotropic structure.

### 3.4. Compression Deformation Test

**Figure 6:**
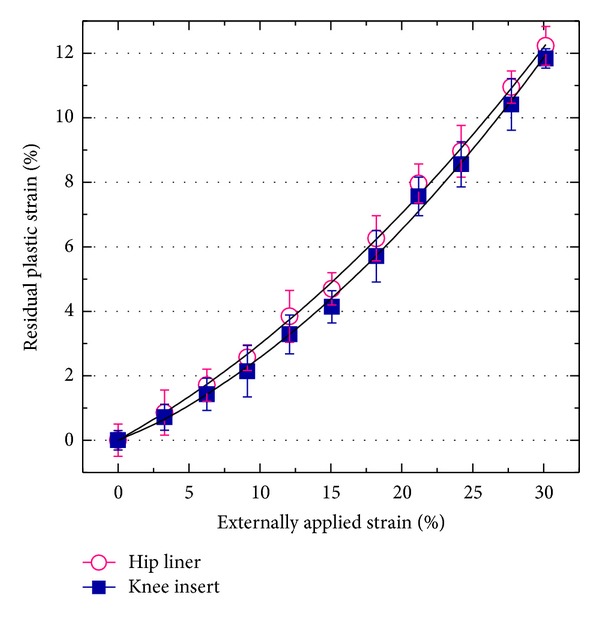
Experimental plots of the compressive deformation and shape-recovery behavior of the as-received XLPE hip and knee components.

Figure 6 shows the plots of residual plastic strain (*ε*_*f*_) as a function of externally applied strain (*ε*_*i*_) for the hip and knee bearings. The full lines represent the fitting curves to the experimental data. The strain behaviors of these components before and after shape recovery were found to phenomenologically obey the quadratic functions as follows:

(7) $$\begin{array}{c} \varepsilon_{f}=0.0054{\varepsilon_{i}}^{2}+0.2440\varepsilon_{i},\;\left(R^{2}=0.999\right),\\ \varepsilon_{f}=0.0067{\varepsilon_{i}}^{2}+0.1983\varepsilon_{i},\;\left(R^{2}=0.998\right), \end{array}$$

for XLPE hip and knee prostheses, respectively. This experimental data suggests that the microstructure of the knee insert has a slightly higher capacity of shape recovery than the hip insert despite the lower amount of radiation, that is, a lower residual plastic strain (*ε*_*f*_) accumulation in the knee insert.

## 4. Discussion

Confocal/polarized Raman spectroscopy allows quantitative and rigorous assessments of the three-phase percentages and crystallographic texture of XLPE hip and knee implants. It was clearly confirmed that all the microstructural parameters investigated in this study showed a transitional behavior from the surface down to the subsurface regions (cf. Figures 3–5). The phase percentages rapidly change within the first 35 *μ*m below their surfaces and the lowest crystallinity (*α*_*c*_) was always detected in the very surfaces. Correspondingly, molecular chain orientation parallel to the articulating surface with high anisotropy was also observed within those regions. The above structural characteristics can be considered the main effects of decrystallization induced by surface finishing performed at the end process of manufacturing, that is, lathe grinding and polishing. According to our past study using Raman spectroscopy, the original bulk *α*_*c*_ of GUR 1050 resin was reported as ≈50% in its nonirradiated and nonheated state [36]. Considering the *α*_*c*_ values detected in the bulk region of XLPE hip liner (*α*_*c*_ = 45.04 ± 1.15%), a ≈5 vol.% loss of crystalline phase can be identified as a result of its fabrication processes. The gamma irradiation by itself might possibly destroy the crystalline regions at certain high doses but the 100 kGy irradiation does not substantially influence crystallinity change [37, 38]. Thus, it mostly appears to be due to the postirradiation remelting. Although remelting is highly beneficial from the perspective of oxidation resistance [38], the present results indicate that it comes at the price of the reduced crystallinity which is induced by heating above the melting temperature. On the other hand, it was reported that the as-received resin of GUR 1020 initially possessed about 6% a higher *α*_*c*_ over the GUR 1050 [35], but XLPE knee insert eventually showed a comparable *α*_*c*_ level to the analyzed hip liner, indicating a total of ≈11 vol.% loss of crystalline phase during its remelting. Spiegelberg et al. [39] reported that since GUR 1020 has a higher polydispersion index, its efficiency of cross-linking was rather lower than GUR 1050 under the same dose level. Thus, the irradiated GUR 1050 has lower degree of freedom in molecular motion due to more topologically constrained (cross-linked) structure networks. In other words, the irradiated GUR 1020 resin with lower molecular weight as well as lower cross-link density can possess higher mobility and activity of molecular chains, possibly resulting in the increased susceptibility to thermal treatment and consequently in greater decrystallization during remelting.

**Figure 7:**
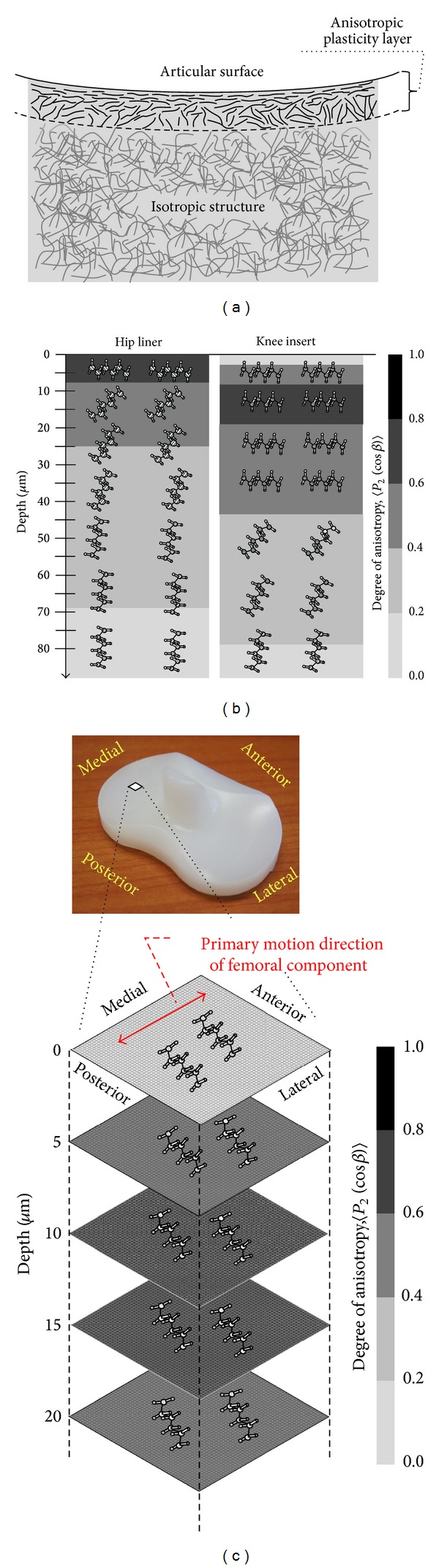
Schematic of the as-received XLPE microstructure consisting of anisotropic plasticity layer and isotropic random structure (a), the enlarged cross-section view of the near-surface anisotropic layers indicating the preferential tilt angles of molecular chains and the degree of crystalline anisotropy in the as-received XLPE hip and knee components (b), and the enlarged longitudinal sectional view of the near-surface anisotropic layers indicating the preferential inplane angles of molecular chains and the degree of crystalline anisotropy in the as-received XLPE knee component (c).

As illustrated in Figures 3–5, the investigated implants showed marked differences in the near-surface morphologies by contrast to the similarities on the bulk microstructure. A slightly lower crystallinity was detected in the knee surface as compared to the hip surface. In addition, it was clearly confirmed that the surface anisotropy was low in the knee but high in the hip. The above structural features might imply the technological differences in each machining procedure adopted for producing the geometrically different shape components of the hip and knee joint prostheses. Microstructural models for summarizing the preferred chain orientation and anisotropic features were given in Figures 7(a)–7(c) for each product. As shown in Figures 7(a)-7(b), the ≈100 *μ*m thick anisotropic layer with transitional morphologies was located on the isotropic region which accounts for a large part (≈99%) of the overall thickness of each component. The anisotropic surfaces with the decreased crystallinity are a clear structural evidence for initial existence of plasticity layer formed in their manufacturing stages. It can be considered that the above two phases of the anisotropic surface and isotropic bulk structures are mainly responsible for wear and creep resistance, respectively. The mechanical properties of polyethylene are governed by (i) molecular weight (ii) balance among amorphous, crystalline, and third intermediate phase percentages; (iii) preferred molecular orientation; (iv) degree of crystalline anisotropy; (v) degree of cross-linking; and (vi) presence or absence of additives. The tested two prostheses had no preferential orientation in their bulks and contain no additives such as calcium stearate and antioxidant agent. Although, as explained above, the knee insert can possess a less cross-link density, it showed a slightly higher capacity of shape recovery, that is, a less accumulation of residual strain, against the applied uniaxial compressive load (cf. Figure 6). It was reported that a higher molecular weight GUR 1050 had greater elastic properties and higher resistance to permanent deformation over GUR 1020 in the as-received states [40]. In principle, the driving force of polyethylene shape recovery is the elastic force associated with the relaxation of amorphous phase having a physical entanglement, that is, cross-linking. This phase is known to be highly recoverable in a rubber-like manner after removal of the applied compressive load [34, 41]. As far as the above-mentioned structural features are concerned, a markedly higher percentage of amorphous bulk in the knee insert (cf.  Figure 3(a)) can be deemed to contribute to a slightly higher efficiency of recovery against the same magnitudes of the applied strain despite its possibly less cross-linking within the amorphous regions. Nevertheless, since this phase is the most easily to be distorted within the structures during an application of ongoing compression, the amorphous-phase-rich knee insert might be less resistant to a time-dependent (creep) deformation under the same magnitudes of external stresses than the hip liner. Our previous result of strain recovery test conducted at the same condition for different types of thermally treated XLPEs [42] showed a linear dependence between *ε*_*i*_ and *ε*_*f*_. The currently observed quadratic dependence of *ε*_*i*_-*ε*_*f*_ plots can partly be interpreted as a consequence of higher percentages of the noncrystalline regions.

For the wear resistance viewpoints, scientific links between frictional characteristics and anisotropic structure had long been studied in conventional (nonhighly cross-linked) UHMWPE under lubricated and unlubricated dry sliding conditions [19, 20, 43]. It is well recognized that, under multidirectional stress fields generated by joint articulation, a highly anisotropic polymer surface leads to a decreased resistance to adhesive wear due to frequent occurrence of strain-softening and weakening even in absence of third-body particles [19, 20, 43]. This micromechanical phenomenon was originally based on the finding of Ramamurti et al. [21] and Wang et al. [19] in which there was a crossing of multidirectional shear forces at intersection of wear paths on femoral head surface. Thus, these observations provided with the perspective that a polyethylene hip liner with highly anisotropic surface can be susceptible to shear forces and wear damage due to an occurrence of sliding motion in a direction traverse to preferred orientation of molecular chains. However, unlike in the case of conventional UHMWPE, the introduction of cross-linking can mitigate adverse impacts of surface anisotropy on wear resistance due to the increased inter- and intralamellar covalent bonds. The validity of such positive effects of cross-linking has already been ascertained by hip joint simulator study under nonabrasive/abrasive conditions demonstrating a significant reduction in volumetric wear for XLPE liner compared to non-cross-linked liner [44] despite having high anisotropy on its surface (cf. Figure 5(c)). Although the predominant factor deciding the wear resistance of XLPE hip liner would be the cross-link density, a surface treatment to reduce its initial anisotropy (e.g., a change in machining conditions) may have a potential clinical benefit in further improving the wear resistance.

On the other hand, Wang et al. [19] pointed out that the strain-softening would not be severe even under combined knee motions of flexion/extension and internal/external rotation because of small difference (<20%) of maximum shear stress component for a knee joint between traverse and longitudinal direction. It is hence considered that strain-softening is more directly relevant to a hip-joint kinematics rather than a knee joint. However, according to the inplane orientation model shown in Figure 7(c), strain-softening is actually still a possibility for the knee due to the existence of the oriented chains orthogonal to the primary-motion direction of femoral component, that is, anterior to posterior direction. The previous joint-simulator study found a much higher sensitivity to abrasion wear in XLPE knee insert than in XLPE hip liner [44]. This fact could be partly associated with the detrimental effects of the surface oriented chains orthogonal to the primary motion on the knee wear. Therefore, a careful consideration should be given to the negative effects of surface molecular orientation on wear behavior even though the polyethylene prosthetic surface has a highly cross-linked microstructure. In the above contexts, our present morphological observations can highlight the importance for XLPE prostheses to optimize by different technological regimes (e.g., reconsideration of machining conditions or techniques) the spatial arrangement of initial molecular orientation relative to the primary directions of motion at each joint for further maximizing their wear performances.

## 5. Conclusion

The quantitative and nondestructive analyses of confocal/polarized Raman spectroscopy were applied to describe and compare the depth profiles of the three-phase percentages and crystalline anisotropy between XLPE hip and knee replacement implants of the first-generation highly cross-linked ultrahigh molecular weight polyethylene. The impacts of the surface and subsurface morphologies on plastic deformation and wear were explicitly discussed from the viewpoints of the molecular mobility and strain-softening resistance. The main outcomes of our investigation can be summarized as follows.

- After the procedure of remelting, a higher loss of crystallinity percentage was observed in the irradiated GUR 1020 resin used for the knee insert, as compared to the irradiated GUR 1050 for the hip liner.
- It was confirmed that the studied hip and knee components consisted of two structural regions induced by the manufacturing procedures: the near-surface transitional anisotropic layer (≈100 *μ*m thickness) and the bulk isotropic structural region.
- The knee insert showed a slightly higher capacity of the shape recovery against the applied uniaxial compressive load over the hip liner owing to a markedly higher percentage of the bulk amorphous phase with cross-linking.
- Our present observations implied the possibility to further maximize their wear performances by the surface rearrangements of crystalline texture as the outcome of different technological regimes for surface machining and polishing during the production processes.
